# Immunohistochemical Evaluation of Notch1 in Ovarian Tumours and Its Prognostic Implications

**DOI:** 10.7759/cureus.40830

**Published:** 2023-06-22

**Authors:** Varsha Ginnavaram, Arumugam Vasugi, Sandhya Sundaram

**Affiliations:** 1 Pathology, Sri Ramachandra Institute of Higher Education and Research, Chennai, IND

**Keywords:** notch1, ovarian cancer, immunohistochemistry, targeted therapy, prognosis

## Abstract

Background: Ovarian cancer is the fifth most common malignancy among women and the second most common gynaecological malignancy. Surface epithelial ovarian tumours constitute two-thirds of all ovarian tumours. Most high-grade serous carcinoma patients gain an initial complete response but eventually succumb to relapse and death leaving the overall survival grim. Therefore, new regimens targeting the pathways involved in metastasis and chemoresistance are essential for the development of more effective therapies. Notch signalling is one of the pleiotropic signalling pathways that plays a key role in differentiation and tissue morphogenesis. It has been observed that this Notch signalling pathway is seen to be deregulated in various cancers. It is thought to have an oncogenic role in ovarian cancer. Our objective in this study is to evaluate the immunohistochemical expression pattern of Notch1 in surface epithelial ovarian tumours and its correlation with the clinicopathological profile.

Methods: This study includes a total of 100 cases of borderline and malignant surface epithelial ovarian tumours. Clinical data of the patients were obtained from the medical records section. HPE slides were examined and one representative paraffin block was selected for each patient. IHC of Notch1 was performed and analyzed. The staining pattern for Notch1 was calculated using the Q score.

Results: Immunohistochemistry (IHC) evaluation of Notch1 in surface epithelial ovarian tumours in this study showed an increased intensity of Notch1 staining in high-grade serous malignant tumours. The grading and staging of tumours were compared with Notch1 expression. Statistical analysis was performed using Spearman Rank order correlation analysis. There was a significant correlation (0.01 level, two-tailed) between the grading and staging of ovarian tumours and Notch1 expression.

Conclusion: Assessing Notch1 expression in ovarian cancer by IHC is a useful tool in view of its clinical applications, development of targeted therapies and as a marker of prognosis. The intensity of the Notch1 stain appears to be directly proportional to the grade of tumour. This may offer a potential targeted therapy against the Notch signalling pathway in tumours that strongly express Notch1.

## Introduction

Ovarian tumours are more varied compared to any other organ as they inherit a wide array of histogenetic backgrounds. Ovarian cancer (OC) is on the rise being the fifth most common cancer among women and ranking seventh as the cause of cancer deaths worldwide. According to Indian registries, the ovaries are the third leading site of cancer among women following cervical and breast cancer. About 80% of ovarian tumours are benign, which occur commonly in young women between 20 and 45 years whereas borderline tumours occur at slightly older age. The incidence of malignant ovarian tumours increases with age, occurring in pre-menopausal and perimenopausal women. Epithelial ovarian tumours account for approximately two-thirds of all ovarian tumours and their malignant forms represent 90% of all OCs, with 10% comprising other tumours like germ cell tumours and stromal tumours [1].

The pathogenesis of ovarian tumours is believed to be influenced by several prevailing theories, including repeated ovulation with associated tissue trauma, heightened oestrogen levels stemming from excess gonadotropin release, elevated androgen levels, and stromal hyperactivity. The older theories suggest that ovarian surface epithelial cell gives rise to these cancers. But the scientific work of the past decade has proposed that high-grade epithelial ovarian carcinomas cannot be considered a single disease entity owing to various findings two of such being the detection of TP53 mutations and serous tubal intraepithelial carcinoma (STIC) lesions in association with familial BRCA mutations suggesting that cancer cells are shed from the tubal epithelium and implanted on the surface of the ovary [2].

The exact molecular transformation events causing OC are not known. It is thought that various mutations in genes and alterations in signalling pathways by ovarian epithelial stem cells promote the development and progression of OC. A few of these signalling pathways include TGF-Beta, Wnt signalling pathway, Notch signalling pathway, NF-kB signal transducer and transcriptional activator 3 (STAT3) pathway and Hedgehog pathway. These pathways directly or indirectly regulate epithelial-mesenchymal transition. Notch signalling is thought to influence the ovarian germline stem cells and plays an important role in deciding cell fate, proliferation and differentiation. It is also thought to have an influence on cell-to-cell interactions. However, the role of the Notch pathway in the mammalian ovary is still unknown [3]. These molecular alterations in epithelial ovarian tumours can be studied using immunohistochemistry (IHC) staining for Notch1 in the tumour cells, based on the various staining patterns and correlating the same with the grade and other parameters. The aim of the present study is to analyze the role of the Notch1 pathway in epithelial ovarian tumours and correlate the expression pattern with clinicopathological parameters and assess the difference in the expression of Notch1 between borderline and malignant surface epithelial ovarian tumours.

## Materials and methods

A total of 100 cases of histopathologically diagnosed surface epithelial ovarian tumours were retrieved from the surgical pathology files of the Department of Pathology, Sri Ramachandra Institute of Higher Education and Research, Chennai. The samples included ovarian specimens from January 2015 to December 2018. The study was commenced after obtaining permission from the institutional ethics committee (CSP-MED/19/JAN/49/11). Clinical data of the patients including patient age, location of the tumour, laterality, metastasis, and presence of ascites were obtained from the medical records section. The patient identifiers were kept confidential throughout the study. We retrieved and analyzed the histopathological slides stained with hematoxylin and eosin (H&E) for all cases included in our study. One representative paraffin block was selected for each patient based on the inclusion criteria. Metastatic ovarian tumours with primary peritoneal tumours and tumours other than surface epithelial tumours were excluded from the study.

Formalin-fixed paraffin-embedded tissue was used for the study. The sections were cut at a thickness of 4-5 microns, stained with H&E and all the slides were analyzed. Serous carcinoma was graded into a two-tier grading system. Lymph node involvement was assessed and recorded. Pathological staging was done according to the International Federation of Gynecology and Obstetrics (FIGO) staging system. The antibody of interest Notch1 (c-20) (sc-6014) is a goat polyclonal antibody raised against a peptide mapping at the c-terminus of notch1 of human origin. IHC was performed manually on 100 cases of histopathologically proven surface epithelial ovarian tumours in a dilution of 1:100. Carcinoma colon was used as a positive control. For negative control, a non-immunological serum was used instead of the primary antibody. All slides were examined and the staining pattern for Notch1 was calculated using the Q score (Table 1). The extent of the IHC stained area was scaled as 0 for no IHC signal at all, 1 for <10%, 2 for 10-50%, and 3 for >50% of tumour cells. The score for IHC intensity was also scaled as 0 for no IHC signal, 1 for weak, 2 for moderate and 3 for strong IHC signals. The final score used in the analysis was calculated by multiplying the extent score and intensity score with a minimum score of 0 and a maximum score equal to 300. Results are scored by multiplying the percentage of positive cells (P) by the intensity (I). Formula: Q=PxI.

**Table 1:** IHC staining analysis using Q score IHC: immunohistochemistry

			Extent of IHC staining					
Score		0		1		2	3	
Positive cells (p)		0		<10%		10-50%	>50%	
	Intensity of IHC staining							
Score	0				1	2		3
Positive cells (I)	No staining				Weak	Moderate		Strong

Statistical analysis

Statistical analysis was performed using the Spearman Rank Order correlation coefficient. Variables following normal distribution were expressed as mean (standard deviation), and variables that followed skewed distribution were expressed as a median. Grading and staging of ovarian tumours were ordinal data. A Notch1 score is a scale data. Spearman Rank Order Correlation analysis was performed to compare the grading and staging of ovarian tumours with Notch1 expression. The p-value of < 0.05 is considered as being statistically significant.

## Results

Patient characteristics

**Figure 1:**
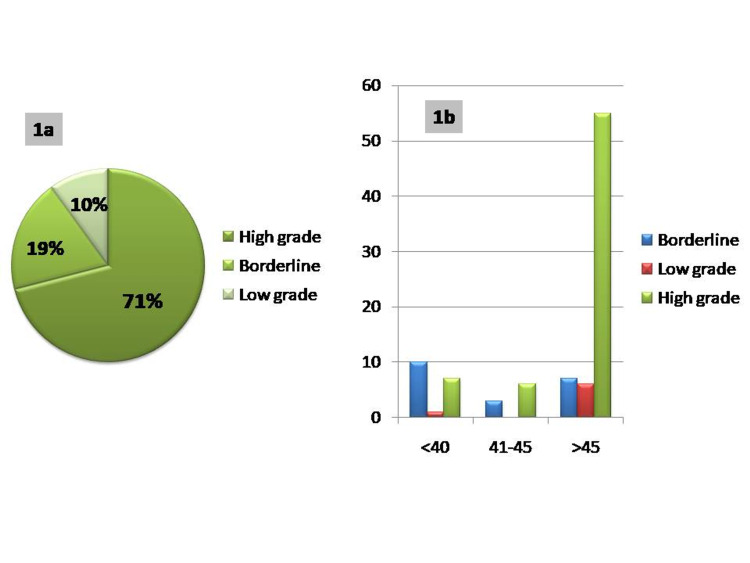
Pictograph Figure 1a: Frequency of borderline, low grade and high-grade tumours. Figure 1b: Age distribution in borderline, low grade and high-grade tumours.

**Figure 2:**
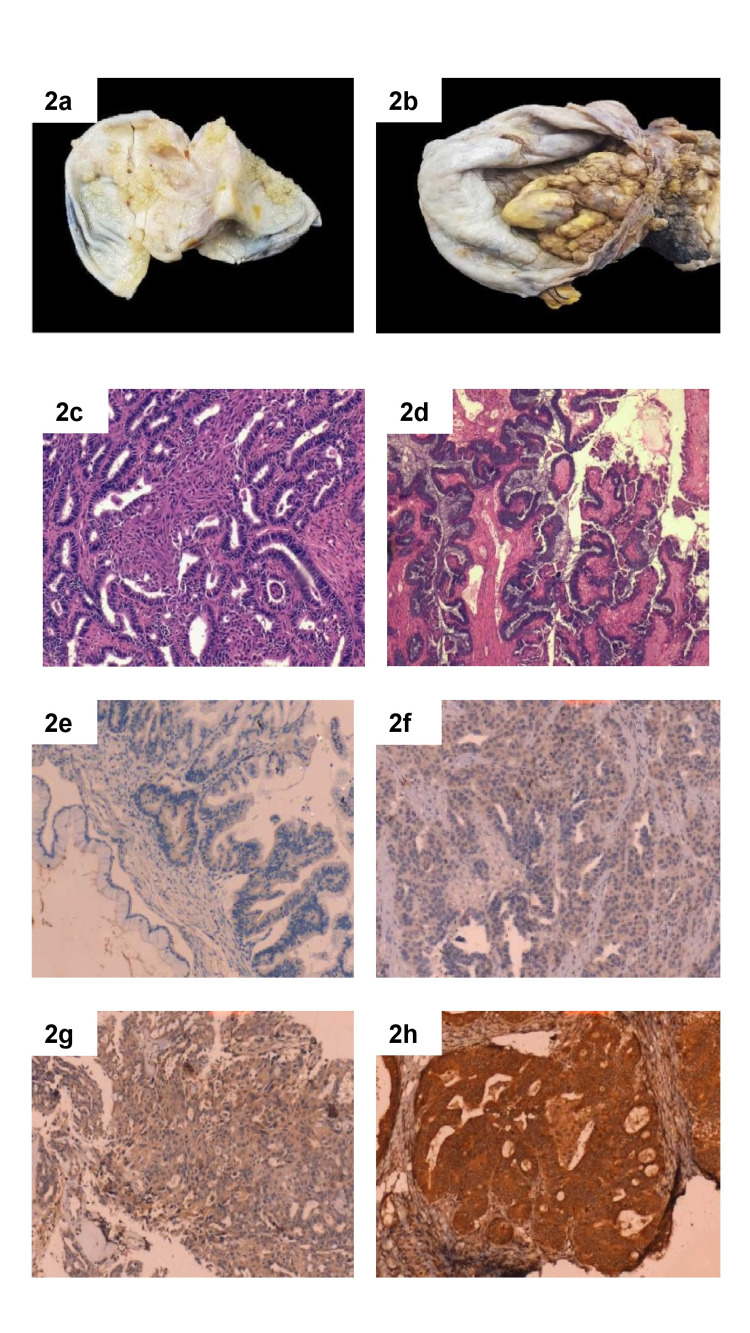
Gross, Histopathology, Immunohistochemistry Figure 2a: Gross of serous papillary carcinoma showing cyst with papillary areas. Figure 2b: Gross of clear cell carcinoma showing solid and cystic areas. Figure 2c: Histopathology of high-grade serous carcinoma showing infiltrating tumour cells in papillary architecture (H&E x100x). Figure 2d: Histopathology of mucinous carcinoma (H&E x 100x). Figure 2e: Immunohistochemistry of negative Notch1 (IHC x 100x). Figure 2f: Immunohistochemistry of Notch1: 1+ (IHC x 100x). Figure 2g: Immunohistochemistry expression of Notch1: 2+ (IHC x 100x). Figure 2h: Immunohistochemistry expression of Notch1: 3+ (IHC x 100x). IHC: Immunohistochemistry, H&E: hematoxylin and eosin

Of the 100 cases, there were 19 borderline tumours (19%), 10 low grade (10%), and 71 high grade (71%) surface epithelial ovarian tumours which included serous, mucinous, seromucinous, endometrioid, Brenner and clear cell tumours (Figure 1a). Ovarian tumours were present in all age groups. The age ranged between 17 and 85 years in the present study (Figure 1b). The youngest patient in this study was a 17-year-old girl reported with left-sided low-grade serous carcinoma. The oldest patient was an 85-year-old lady with left ovarian endometroid carcinoma. The mean age is 51 years. The patients were divided into three age groups as follows: (a) reproductive age group: 14 to 40 years; (b) perimenopausal age group: 41-45 years; (c) post-menopausal age group: >45 years. The incidence of malignant tumours was found to be higher in the post-menopausal age group. Only 26 out of 100 tumours had a bilateral presentation. Among bilateral tumours 25 (96.15%) were malignant. Grossly, the tumours were cystic, solid or both solid and cystic in nature. In the present study, 26 out of 100 (26%) tumours were purely cystic. Solid tumours were seven out of 100 (7%). The combined solid and cystic presentation was present in 67 tumours (67%) (Table 2) (Figure 2a, 2b, 2c, 2d).

**Table 2:** Patient characteristics

Patient Characteristics (100 cases)			
1. Age distribution	14-40 yrs	41-45 yrs	>45 yrs
	20 (20%)	11 (11%)	69 (69%)
2. Tumour types	Low grade	Borderline	High grade
	10 (10%)	19% (19%)	71% (71%)
3. Gross morphology	Cystic	Solid and cystic	Solid
	26 (26%)	67 (67%)	7 (7%)

Ovarian tumour characteristics

In our study, all the cases were staged according to the 2016 WHO: TNM staging of tumours. The majority of the cases of serous carcinoma belong to p T 1a- 37% (tumour limited to one ovary, capsule intact, no malignant cells in ascitic fluid); it is followed by p T 1c by 15% (tumour involves one or both the ovaries with either surgical spill, capsule rupture or positive peritoneal wash). In the current study, we report 13% of malignant cases that showed nodal positivity (p N1) and 57% of the cases did not have any nodal involvement and for 30%, lymph node status could not be assessed. Only 2% of the tumours showed distant metastasis and the remaining 98% did not show any metastasis. Out of 81 malignant cases, malignant cells in the ascitic fluid were present in 12 cases. All the cases were staged according to the 2016 FIGO staging of ovarian tumours. Among malignant tumours, the greatest number of cases were reported in stage I (44%) and stage III (26%). Among borderline tumours, 20 were stage I and one case was stage III. There is a correlation between FIGO staging and laterality. All the stage 4 tumours were bilateral. No significant correlation was found between staging and other histological parameters.

Notch1 expression

All 100 cases were subjected to IHC staining with Notch1 antibody (Figure 2e, 2f, 2g, 2h). Out of 19 borderline cases, nine cases were negative and 10 showed a score of 1+. Out of 19 borderline cases, four cases of serous borderline tumours showed 1+ and four cases were negative for Notch1. Out of mucinous borderline tumours, six showed 1+ staining and four cases were negative. One case of seromucinous borderline tumour was negative for Notch1. Among 81 malignant cases, six cases were negative for Notch1, 14 cases were 1+, three cases showed 2+ and 58 cases showed 3+. Malignant cases in the present study showed predominantly 3+ staining. Among serous carcinoma, 45 cases showed 3+ staining, eight cases showed 2+ and three cases were negative. Out of eight cases of mucinous carcinoma, five showed 2+ staining and three showed 3+. Endometrioid carcinomas predominantly showed 3+ staining. Two out of three clear cell carcinomas showed 3+ staining and one case was 2+. Malignant Brenner tumour showed 3+ staining.

The subjective outcomes of the cancer prognosis were evaluated using the NOTCH score and the cancer grade. The grading and staging of tumours were compared with Notch1 expression. Statistical analysis was performed using Spearman Rank order correlation analysis. There was a significant correlation (0.01 level, two-tailed) between the grading and staging of ovarian tumours and Notch1 expression (Table 3 and Table 4).

**Table 3:** Statistical analysis correlating Notch1 score and stage of ovarian tumours

Correlations				
			NOTCH Score	Stage of the tumour
Kendall's tau_b	NOTCH Score	Correlation Coefficient	1.000	.346^**^
		Sig. (2-tailed)	.	.000
		N	100	100
	Stage of the tumour	Correlation Coefficient	.346^**^	1.000
		Sig. (2-tailed)	.000	.
		N	100	100
Spearman's rho	NOTCH Score	Correlation Coefficient	1.000	.421^**^
		Sig. (2-tailed)	.	.000
		N	100	100
	Stage of the tumour	Correlation Coefficient	.421^**^	1.000
		Sig. (2-tailed)	.000	.
		N	100	100
**. Correlation is significant at the 0.01 level (2-tailed).				

**Table 4:** Statistical analysis correlating Notch1 score and grade of ovarian tumours

Correlations				
			Grade of the Tumour	NOTCH Score
Kendall's tau_b	Grade of the Tumour	Correlation Coefficient	1.000	.688^**^
		Sig. (2-tailed)	.	.000
		N	100	100
	NOTCH Score	Correlation Coefficient	.688^**^	1.000
		Sig. (2-tailed)	.000	.
		N	100	100
Spearman's rho	Grade of the Tumour	Correlation Coefficient	1.000	.794^**^
		Sig. (2-tailed)	.	.000
		N	100	100
	NOTCH Score	Correlation Coefficient	.794^**^	1.000
		Sig. (2-tailed)	.000	.
		N	100	100
**. Correlation is significant at the 0.01 level (2-tailed).				

## Discussion

The mechanistic basis of epithelial ovarian tumours is based on various genetic and epigenetic alterations. Aberrations in cell signalling pathways are implicated in pathogenesis. In correlation with clinicopathological data, we studied the nature of the expression of the Notch1 receptor and alterations in its pathway in epithelial ovarian tumours. Ovarian tumours are seen in all age groups. The age ranged from 17 to 85 years with a mean age of 51 years in the present study. The youngest patient in this study was a 17-year-old girl reported with a left-sided serous carcinoma. The oldest patient was an 85-year-old lady with left endometrioid carcinoma. The incidence of malignant tumours was found to be higher in the postmenopausal age group. These findings were similar to the study done by Brett M et al. [4].

In our study, most of the tumours were unilateral. Only 26 (26%) out of 100 tumours had a bilateral presentation. Among bilateral tumours 25 cases (96.15%) were malignant. Thus, most malignant tumours have a bilateral presentation. This finding was in concordance with a study done by R Jha et al. who also reported 42.3% malignant tumours presenting bilaterally [5]. Similar to the present study, the incidence of bilaterality (26.8%) was present in the study by Bell et al. [6]. In the present study, all the metastatic tumours had bilateral presentations similar to a study by Zhao et al. where most of the metastatic tumours have bilateral presentation [7].

Patients with complex ovarian tumours have an increased risk of ovarian malignancy according to a study done by McDonald JM et al. [8]. In our study, grossly the tumours were cystic, solid or both solid and cystic in nature. In the present study, 26 out of 100 (26%) tumours had purely cystic architecture. Solid tumours were seven out of 100 (7%). The combined solid and cystic presentation was present in 67 tumours (67%). In our study 59 malignant cases were solid and cystic in nature. A study by Bell et al. [6] had 58.21% cystic tumours, 13.43% solid and 28.36% combined solid and cystic tumours which could be due to the inclusion of benign cases in their study as most benign tumours have a cystic presentation.

Surface epithelial tumours were classified based on the WHO classification of ovarian tumours. Serous carcinoma was the commonest malignant tumour type constituting 57% of tumours. The second common tumour was mucinous borderline having an incidence of 12%. Other tumour types like serous borderline, mucinous, endometrioid, Brenner and anaplastic were also present. Serous carcinoma was the most common category followed by mucinous carcinomas among malignant tumours while in the borderline tumour category, borderline mucinous tumour was frequent compared to borderline serous tumours. These findings were similar to the study done by Brett M et al. [4]. Out of 100 cases studied, the majority were malignant tumours 81 (81%), and the rest borderline 19 (19%). These findings were in concordance with studies done by Pilli et al. [9].

According to OC incidence statistics [10], the incidence of malignant ovarian tumours is higher in the post-menopausal age group which was similar to our study. The frequency of occurrence of malignant tumours is higher in women belonging to the post-menopausal age groups (71%) and the occurrence of borderline tumours is higher in women belonging to reproductive stages of life (21%). In the premenopausal age groups, the incidence of tumours is comparatively low with malignant being 7% and borderline 1%. A study done by Daniela et al. found an increased incidence of borderline tumours in the reproductive age group over the years but this could be due to the increased efficacy of diagnosing borderline tumours [11]. In the reproductive age group, the majority of the tumours were borderline with malignant tumours contributing to only 11 cases.

According to the study by Ritu Salani et al., the majority of the epithelial tumours present in the advanced stage with distant metastasis and spread to regional lymph nodes [12]. However. In our study majority of the patients (37%) belonged to FIGO stage I (tumour limited to one ovary, capsule intact, or fallopian tube surface or no malignant cells in ascitic fluid) with very few cases showing spread to lymph nodes (13%) and distant metastasis (2%). This could be due to early presentation or early diagnosis in our study population. Malignant ascites are a poor prognostic indicator as ascites are thought to create an environment that forms and mediates protumorigenic signals that can lead to the progression and proliferation of tumour cells. A study done by Nuzhat Ahmed et al. shows that most cases of malignant ascites have distant metastasis and adverse outcome. In our study, both cases that showed distant metastasis were positive for malignant cells in peritoneal fluid [13]. And, 12% of the malignant cases were positive for tumour cells in peritoneal fluid.

Notch is a signalling receptor and its role in tissue homeostasis has been extensively researched. Abnormal expression or silencing of Notch1 can result in tissue abnormalities and the expression of cancer, although the underlying mechanism is poorly understood. Notch has been found to have both oncogenic and tumour suppressor functions depending on the type of malignancy and the subtypes. It was observed that when the expression of Notch is lost in epidermal malignancies, a microenvironment leading to epidermal transformation was created.

According to the literature [3], dysregulation of the Notch pathway is responsible for the initiation and progress of epithelial ovarian tumours. In our study, we found a significant association between the Notch expression and the grading and staging of tumours. The mean Q score showed an increasing trend in relation to the grade of the tumour. This was found to be statistically significant using Spearman Rank Order Correlation analysis with a significant P value (P<0.05 significant). The relationship between the Q score and other parameters like age, laterality, gross appearance, histomorphological type and stage of the tumour did not show any significant correlation. An in-depth study with several downstream markers of the Notch signalling pathway (NOTCH 1- NOTCH 4, DLL 1, 2 & 4 and Jagged 1, Jagged 2) [14] in a larger number of cases should be done to elicit the exact role and clinical significance of notch pathway in epithelial ovarian tumours.

The risk of malignancy associated with various parameters was analysed by employing the Chi-square test and logistic regression analysis in the present study. Few independent variables were identified to evaluate the risk factors to predict malignancy in tumours. Age, menstrual status, tumour laterality, gross appearance, histological features and Notch1 expression were the variables assessed. Out of these risk factors studied, increasing age, postmenopausal status, bilaterality, complex tumour morphology and increased Notch1 score were associated with a higher risk of malignancy. There was no significant correlation between the size of the tumour and the risk of malignancy. Bilateral involvement conferred an increased risk of malignancy compared to unilateral tumours. Complex and solid tumours with papillary tumour morphology were found to be the most important risk factor, predicting an increased risk of malignancy compared to cystic tumours. These findings were in concordance with the study by Darshana S. Patil. As per the study, 92.44% of the cases could be correctly predicted as being malignant and 73.68% of the cases as borderline [15].

## Conclusions

We conclude that the Notch1 signalling pathway plays a significant role in the pathogenesis and progression of epithelial ovarian tumours. The intensity of the Notch1 stain appears to be directly proportional to the grade of the tumour. This may offer a potential therapy targeted against the Notch signalling pathway in tumours that strongly express Notch1. However, determining the protein expression of Notch1 using advanced molecular techniques will enlighten the prognostic and theragnostic use of Notch1 in epithelial ovarian tumours with studies directed towards five and 10-year survival.
